# ABHD5—A Regulator of Lipid Metabolism Essential for Diverse Cellular Functions

**DOI:** 10.3390/metabo12111015

**Published:** 2022-10-24

**Authors:** Margarita Schratter, Achim Lass, Franz P. W. Radner

**Affiliations:** 1 Institute of Molecular Biosciences, NAWI Graz, University of Graz, 8010 Graz, Austria; 2 BioTechMed-Graz, 8010 Graz, Austria; 3 Field of Excellence BioHealth, 8010 Graz, Austria

**Keywords:** ABHD5, CGI-58, ATGL, PNPLA3, triglyceride, lipid metabolism, NLSD, Chanarin-Dorfman syndrome, ichthyosis, NAFLD

## Abstract

The α/β-Hydrolase domain-containing protein 5 (*ABHD5*; also known as comparative gene identification-58, or CGI-58) is the causative gene of the Chanarin-Dorfman syndrome (CDS), a disorder mainly characterized by systemic triacylglycerol accumulation and a severe defect in skin barrier function. The clinical phenotype of CDS patients and the characterization of global and tissue-specific ABHD5-deficient mouse strains have demonstrated that ABHD5 is a crucial regulator of lipid and energy homeostasis in various tissues. Although ABHD5 lacks intrinsic hydrolase activity, it functions as a co-activating enzyme of the patatin-like phospholipase domain-containing (PNPLA) protein family that is involved in triacylglycerol and glycerophospholipid, as well as sphingolipid and retinyl ester metabolism. Moreover, ABHD5 interacts with perilipins (PLINs) and fatty acid-binding proteins (FABPs), which are important regulators of lipid homeostasis in adipose and non-adipose tissues. This review focuses on the multifaceted role of ABHD5 in modulating the function of key enzymes in lipid metabolism.

## 1. Introduction

The α/β-hydrolase domain-containing protein 5 (ABHD5) is a lipid droplet (LD)-associated protein that has been originally identified by a comparative proteomic study of evolutionarily conserved human genes in the nematode *Caenorhabditis elegans* [1]. The protein is therefore also known as comparative gene identification-58 (CGI-58). The human *ABHD5* gene comprises seven exons located on the short arm of chromosome 3. The translation of the 5.37 kb-sized mRNA transcript generates a protein that consists of 349 amino acids with a molecular mass of approximately 39 kDa. Of note, the primary structure of ABHD5 is evolutionarily highly conserved among vertebrates and exhibits an amino acid sequence identity of 94% between the human and murine orthologues. In mice, ABHD5 mRNA is mainly expressed in adipose tissue and testes, but lower levels are also detected in the liver, skin, kidney, and heart [2,3,4].

Loss-of-function mutations in the *ABHD5* gene have been identified as an underlying cause of the Chanarin-Dorfman syndrome (CDS, OMIM:275630), a genetic subtype of neutral lipid storage disease (NLSD) with an estimated prevalence of less than 1:1,000,000. Affected patients suffer from a severe skin disorder known as ichthyosis and exhibit a significant ectopic accumulation of triacylglycerol (TAG) in various tissues including the skin, muscle, liver, nervous system, and blood leukocytes [5,6,7]. Consistent with the clinical manifestation of the disease, ABHD5 has been attributed a fundamental role in neutral lipid metabolism. Extensive research during the past two decades has revealed several functions of ABHD5, including its indispensable role in energy homeostasis by acting as a co-activator of adipose triglyceride lipase (ATGL) [2]. Similarly, ABHD5 has been shown to cooperate with other proteins containing a patatin-like phospholipase domain (PNPLA), such as PNPLA1 and 3, thereby regulating lipid metabolism in the skin and liver, respectively [8,9,10]. In addition, ABHD5 interacts with members of the perilipin (PLIN) and fatty acid (FA)-binding protein (FABP) families, but also binds synthetic and endogenous ligands that regulate protein interaction with PNPLA and PLIN proteins [3,11,12,13,14,15,16,17,18,19]. In this review, we focus on protein interaction partners and ligands of ABHD5 and highlight the functional role of this versatile protein in adipose- and non-adipose tissues.

## 2. Protein Structure of ABHD5

**Figure 1:**
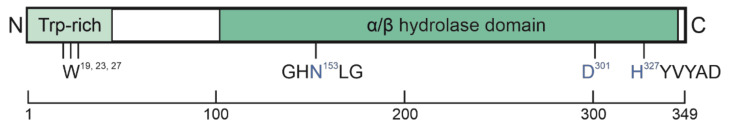
Protein domain organization of human α/β hydrolase domain containing protein 5 (ABHD5). The α/β-hydrolase domain (dark green box), located at the carboxyl terminus of the protein, contains the predicted catalytic triad (indicated in blue) consisting of the consensus GxSxG lipase motif in which the serine is replaced by an asparagine residue (Asn153), and Asp301 as well as His327 within the conserved Hx4D acyltransferase motif. At the amino terminus, ABHD5 harbors a tryptophan (Trp)-rich region (light green box) important for lipid droplet binding.

ABHD5 is a member of the esterase/lipase/thioesterase subfamily of proteins that are structurally defined by the presence of an α/β-hydrolase fold domain [20]. This superfamily includes a variety of enzymes with diverse functions such as proteases, lipases, esterases, dehalogenases, peroxidases, or epoxide hydrolases [21]. In ABHD5, the compact three-layer α/β/α sandwich containing the α/β hydrolase core structure is located at the carboxyl terminus of the protein (Arg102—Glu343 of humane ABHD5, Figure 1) [22]. It consists of eight central β-strands (the first two being antiparallel), flanked by two and four α-helices on each side, respectively. A helical cap region covers the potential binding pocket to prevent the direct access of the active site to the surface. Typically, α/β hydrolase proteins act through a highly conserved catalytic triad consisting of a nucleophilic serine within an esterase/lipase GxSxG motif, an acid, and a histidine residue [20,23]. In ABHD5, however, the catalytic serine residue is replaced by an asparagine residue (Asn153 of humane ABHD5) [24], which most likely eliminates the possibility of the protein to catalytically act as an esterase/lipase [2,12].

The carboxyl terminus of ABHD5 contains the conserved consensus HxxxxD sequence for acyltransferase activity [25]. Seemingly in agreement with this structural feature, Gosh et al. [25] initially reported that ABHD5 in fact acts as an acyl-CoA dependent lysophosphatidic acid acyltransferase (LPAAT) when using recombinant human ABHD5 purified from *Escherichia coli*. This enzymatic activity has then also been reported by Brasaemle and co-workers [26], in line with the hypothesis that ABHD5 may function as an acyltransferase and thus be involved in the generation of the important second messenger molecule phosphatidic acid. However, years later, the same group disproved the LPAAT activity that had been attributed to ABHD5 [27]. They demonstrated that the mutagenesis of the predicted catalytic site for ABHD5 acyltransferase activity did not affect the LPAAT activity of the recombinant purified protein. Instead, the authors clearly showed that the identified LPAAT activity of purified ABHD5 was due to a contamination with the bacterial LPAAT plsC that occurred during the affinity purification process [27]. Thus, ABHD5 does not represent another LPAAT.

At the amino terminus, ABHD5 harbors a partially unstructured, unusually tryptophan-rich region (Met1—Cys45 of humane ABHD5), which appears to be responsible for the correct localization of the protein to cytosolic LDs of adipocytes [22]. So far, no experimental protein structure of ABHD5 has been determined. However, several three-dimensional homology models have been generated [22,28,29]. These computational models provide a useful tool to identify the structural features of ABHD5 and link them to their biological functions.

## 3. Neutral Lipid Storage Disease in Humans—A Disorder with Two Distinct Clinical Phenotypes

Initial evidence that ABHD5 is involved in lipid metabolism derives from a clinical condition known as NLSD, which is associated with massive TAG accumulation in various tissues of the body. The first case report of NLSD was presented by Jordans et al. [30] in the 1950s on two brothers who suffered from severe cardiac and skeletal muscle dystrophy. The affected patients accumulated a large number of TAG-containing LDs in various tissues, including cardiac and skeletal muscle, liver, nervous system, and peripheral blood granulocytes. The latter condition was termed Jordans’ anomaly, which now represents an important diagnostic marker of NLSD. In 1966, Rozenszajn and colleagues [31] reported additional cases of NLSD patients who exhibited Jordans’ anomaly but, interestingly, showed no evidence of cardiac or skeletal myopathy. Instead, the patients developed severe ichthyosis, a clinical symptom characterized by an impaired skin barrier function and excessive and visible scaling that typically affects the entire skin’s surface [32]. Later, in the 1970s, Dorfman et al. [7] and Chanarin et al. [5] provided additional case studies of NLSD patients with ichthyosis, and the disorder was subsequently termed CDS. From these initial NLSD case reports, it became evident that the clinical manifestation of the disorder is very heterogeneous [33]. Accordingly, NLSD patients are now classified in two groups depending on whether the patient suffers from ichthyosis (NLSD with ichthyosis, NLSDI/CDS) or instead develops a severe form of skeletal and/or cardiac myopathy (NLSD with myopathy, NLSDM, OMIM:610717), a condition not found in NLSDI patients.

Although the clinical phenotype of NLSD had already been known for decades, the causative genes for the disease’s pathogenesis remained elusive for a long time. In 2001, Judith Fischer’s group described loss-of-function mutations in the ABHD5 gene in 13 patients from nine families suffering from NLSDI [24]. Since then, the number of identified ABHD5 mutations has steadily increased, including common gene variations such as deletions [24,34,35], insertions [24,36,37,38,39], missense [24,40,41,42], nonsense [2,36,40,43], and splice-site mutations [24,34,35,44,45], but also unusual mutations such as large genomic deletions [35,46] or an intronic insertion of a transposable LINE-1 element leading to aberrant *ABHD5* mRNA splicing [37]. Interestingly, the reported common NLSDI mutations are distributed across the entire *ABHD5* gene [47]. To date, around 140 clinical NLSDI cases have been described worldwide; most of them from the Mediterranean region and the Middle East, but cases have also been reported from Japan, China, India, and Brazil in the literature [47]. All patients suffer from ichthyosis and exhibit the typical Jordans’ anomaly and systemic TAG accumulation. More variable clinical manifestations of NLSDI include liver steatosis with hepatosplenomegaly, neurological and developmental disorders comprising cataracts, ectropion, growth retardation, small ears, ataxia, hearing loss, horizontal nystagmus, and mental retardation, as well as, in rare cases, skeletal myopathy [48].

**Figure 2:**
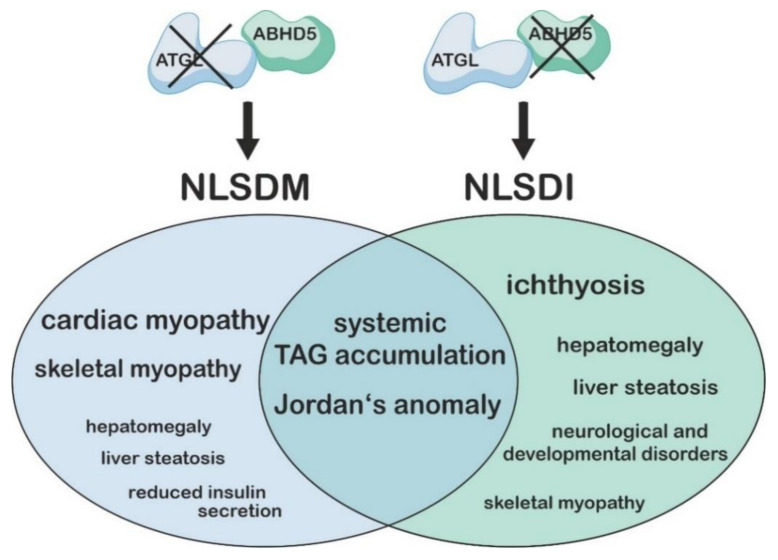
Similarities and differences in the clinical manifestations of Neutral Lipid Storage Disease with Myopathy (NLSDM) vs. Neutral Lipid Storage Disease with Ichthyosis (NLSDI). Mutations in the gene encoding adipose triglyceride lipase (ATGL) cause NLSDM, while mutations in the gene coding for α/β hydrolase domain containing protein 5 (ABHD5) are associated with NLSDI. Both syndromes are characterized by systemic triacylglycerol accumulation and Jordans’ anomaly, which describes an accumulation of lipid-containing vacuoles in leukocytes. While NLSDM is associated with severe forms of cardiac myopathy, patients with NLSDI always suffer from ichthyosis. In addition, NLSDM patients often show clinical manifestations of skeletal myopathy and less commonly hepatomegaly, liver steatosis, or reduced insulin secretion rates. In contrast, NLSDI patients often suffer from liver steatosis, hepatomegaly, and neurological disorders and rarely from skeletal myopathy.

Patients corresponding to the NLSDM clinical phenotype do not exhibit mutations in the *ABHD5* gene. Instead, in 2007, Fischer and colleagues identified four different mutations in the human *PNPLA2* gene (encoding for ATGL) in three NLSDM patients [49]. Since then, more than 30 different mutations in *PNPLA2* that are causative for the development of NLSDM have been described [50,51,52,53]. Most of these are nonsense mutations that introduce premature stop codons, resulting in truncated versions of ATGL. Similar to ABHD5 deficiency, the loss of functional ATGL in humans results in Jordans’ anomaly and systemic TAG accumulation, but less commonly in hepatic steatosis or hepatosplenomegaly, and never in neurological or developmental disorders [48]. The similarities and differences in the clinical manifestations of NLSDI and NLSDM are summarized in Figure 2 and imply that ABHD5 and ATGL exert distinct functions in regulating epidermal, hepatic, and cerebral lipid metabolism.

## 4. ABHD5 Is Involved in the Mobilization of Intracellular TAG Stores

In order to explore the corresponding biochemical defect associated with NLSDI, several groups performed studies with cells derived from affected patients. These early biochemical experiments showed that TAG content increased up to 20-fold in dermal fibroblasts from NLSDI patients compared to control cells obtained from healthy individuals, even when the cells were cultivated in lipid-depleted media [54]. Further studies showed that the excess of lipid was stored in cytoplasmic LDs and that acid lipase activity was not altered in the NLSDI cells excluding a defect in lysosomal degradation pathways [6,55,56,57]. Likewise, the TAG accumulation in NLSDI fibroblasts could not be explained by changes in FA uptake, transport, or β-oxidation [54]. In addition, neither the activities of enzymes for glycerophospholipid synthesis [58,59] nor those of lipases [54,57,59] were altered in the NLSDI cells. Defects in ether lipid [59] or cholesterol [6,60] metabolism were excluded as underlying causes for the massive TAG accumulation in patient cells. However, the experiments performed by Salvayre et al. [57] and Williams et al. [59] indicated a deficiency of TAG lipase activity that impairs the degradation of TAGs in NLSDI fibroblasts. Further investigations narrowed it down to a specific defect in the degradation of TAGs containing long-chain FAs and concluded that a long-chain TAG lipase is absent in the NLSDI cells [61]. Quite contrary to these findings, Coleman and colleagues [58,62] reported that the hydrolysis of TAG is not impaired in NLSDI fibroblasts, but that TAG accumulation is caused by rapid TAG re-synthesis. In pulse-chase experiments in combination with the acyl-CoA synthetase inhibitor triacsin C, they found that this was due to a defect in the recycling of TAG-derived acylglycerols to phospholipids.

## 5. Intracellular TAG Hydrolysis Requires the Presence of ABHD5

The biochemical functions of ABHD5 in lipid and energy metabolism remained elusive until 2006 when Lass and colleagues [2] reported on the specific role of ABHD5 in co-activating ATGL enzyme activity. ATGL belongs to the PNPLA protein family that is structurally characterized by the presence of a highly conserved patatin domain. This protein domain was initially discovered in patatin, the most abundant glycoprotein of the potato tuber, which exhibits nonspecific lipid acyl hydrolase and acyltransferase activities [63]. The patatin domain is composed of a typical three-layer α/β/α hydrolase fold that shares similarities with the canonical α/β hydrolase protein architecture surrounding the catalytic site of numerous hydrolases [64]. It contains the active site serine residue within the consensus GxSxG lipase motif that is part of a catalytic Ser-Asp dyad (Ser47 and Asp166 of human ATGL) [65].

The human genome harbors nine genes encoding PNPLA proteins (PNPLA1—9) [65,66]. *PNPLA2* encodes for ATGL and is located on the chromosome 11p15.5 spanning ten exons. The translation of the *PNPLA2* mRNA transcript generates a 504 amino acid protein that shares 86% of its sequence identity with its mouse orthologue [67]. ATGL is highly expressed in white and brown adipose tissues, and to a lesser extent in cardiac and skeletal muscle [67,68]. In 2004, three groups made an important discovery when they independently identified ATGL as a very specific hydrolase with a substrate preference for TAG containing long-chain FAs [67,69,70]. It was found that the enzyme catalyzes the initial and rate-limiting step of a process commonly referred to as intracellular lipolysis [71]. This central pathway in lipid and energy metabolism mobilizes FAs from TAG molecules stored in intracellular LDs for use as energy substrates, cellular signaling molecules, or building blocks of biological membranes. For the complete degradation of TAG to glycerol and FAs, three major hydrolytic enzymes act sequentially, each releasing one molecule of FA from TAG: (i) ATGL specifically removes the first FA of TAG, thereby generating diacylglycerol (DAG) [67]; (ii) which in a second step serves as substrate for hormone-sensitive lipase (HSL), leading to the formation of monoacylglycerol (MAG) [72,73]; (iii) and in the final step of lipolysis, MAG lipase (MGL) degrades MAG to glycerol and FA [74,75]. During lipolysis, ATGL requires the presence of ABHD5 to efficiently hydrolyze TAG. In an elegant study, Lass and colleagues [2] showed that ABHD5 specifically interacts with ATGL and stimulates its TAG hydrolase activity many-fold in vitro. Consistent with these findings, the mutant variants of ABHD5 p.Gln130Pro, p.Glu260Lys, and p.Gln190Ter, as described in NLSDI, failed to activate ATGL [2], which demonstrated that the defective stimulation of ATGL by ABHD5 is causative for the multi-systemic lipid accumulation in NLSDI patients. Conversely, the expression of functional ABHD5 in dermal NLSDI fibroblasts restored the TAG hydrolase activity and reversed the lipid storage phenotype in patient cells [2]. This study provided crucial insights into the pathogenesis of NLSD and established ABHD5 as a key regulatory factor in neutral lipid metabolism. A few years after this important discovery, the G0/G1 switch gene 2 (G0S2) [76,77] and the hypoxia-inducible lipid droplet-associated protein (HILPDA) [78,79] were identified as potent endogenous inhibitors of ATGL, revealing additional mechanisms of the posttranslational regulation of ATGL activity that control lipolysis and FA availability [80].

**Figure 3:**
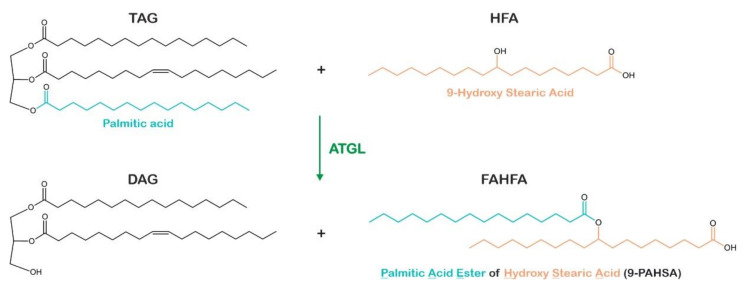
Generation of branched fatty acid esters of hydroxy fatty acids by transacylase activity of adipose triglyceride lipase. Adipose triglyceride lipase (ATGL) catalyzes the transfer of fatty acids (FAs, FA chain colored in blue) from triacylglycerol (TAG) or diacylglycerol (DAG) molecules onto hydroxy fatty acids (HFAs, HFA chain colored in orange), resulting in fatty acid esters of hydroxy fatty acids (FAHFAs).

Interestingly and in addition to its TAG hydrolase activity, ATGL also exhibits acylglycerol transacylase activity, using MAG as acyl donor and MAG or DAG as acyl acceptors to synthesize DAG and TAG, respectively [69,81]. The physiological relevance of this activity has remained elusive. However, Barbara Kahn’s group [82] recently demonstrated that ATGL is involved in the synthesis of branched FA esters of hydroxy FAs (FAHFAs). These lipids belong to the group of mono- or oligomeric FA esters, named estolides, which exhibit anti-inflammatory and anti-diabetic effects in mice and humans [83]. The authors showed that ATGL catalyzes the formation of an ester bond between HFAs and FAs derived from TAG or DAG to generate FAHFAs (Figure 3) [82].

**Figure 4:**
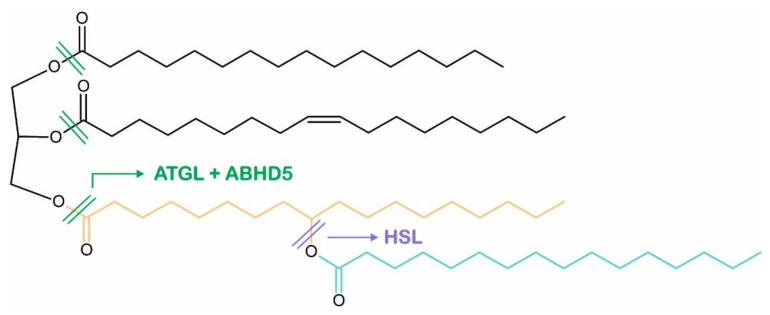
Molecular structure of triacylglycerol estolide. Adipose triglyceride lipase (ATGL, co-activated by α/β hydrolase domain containing protein 5, ABHD5) and hormone-sensitive lipase (HSL) show different substrate specificities towards ester bonds in triacylglycerol estolide molecules as indicated by arrows.

Glycerol-bound FAHFAs in TAG molecules are known as FAHFA-TAGs or TAG estolides and serve as a storage form of bioactive FAHFAs. Recently, Brejchova and colleagues [84] reported that both ATGL and HSL are involved in the catabolism of these lipid stores, but interestingly show different substrate specificity towards TAG estolides and FAHFAs (Figure 4). In the presence of ABHD5, ATGL efficiently hydrolyzes FAHFAs from the glycerol backbone of TAG estolides but is also involved in transesterification reactions and remodeling of TAG estolides, leading to the formation of TAG estolides with alternative acyl compositions. Consistent with these findings, the adipose tissue of ATGL-deficient mice contains decreased levels as well as a reduced variety of TAG estolide species [84]. In addition, we showed that HSL, in contrast to ATGL, readily hydrolyzes the estolide bond of TAG estolides and non-esterified FAHFAs. Consequently, non-esterified FAHFAs and TAG estolides accumulate in the adipose tissue of mice lacking HSL, suggesting a functional role for HSL in the estolide catabolism in vivo [84].

To date, the molecular mechanisms of ATGL activation by ABHD5 are poorly understood, primarily because no experimental three-dimensional structure of any of these proteins exists, nor have the proteins been co-crystallized. Nevertheless, experimental data suggest that the tryptophan-rich region at the amino terminus of ABHD5 is essential for LD binding and a prerequisite for the ability of ABHD5 to stimulate ATGL activity [22]. With regard to this finding, Sanders and colleagues [85] have recently identified two highly conserved amino acid residues of mouse ABHD5 (Arg299 and Gly328) that are crucial for ATGL activation. The mutation of these residues selectively disrupted lipolysis, but did not affect ATGL translocation to LDs, indicating that the activation of ATGL by ABHD5 is dissociable from other ABHD5 functions. In addition, a computational structural analysis revealed a main binding surface of ABHD5 that contains two functionally connected binding pockets [29]. One of these pockets is important for LD targeting and PLIN interaction and the other for ATGL activation. Interestingly, upon co-activation by ABHD5, ATGL broadens its substrate specificity [86]. While the enzyme preferentially hydrolyzes FAs on the *sn*-2 position of TAGs, in the presence of ABHD5, it also cleaves *sn*-1 esterified FAs, generating *sn*-1,3 and *sn*-2,3 DAGs. This stereo- and regioselectivity of ATGL has been shown to be important for the fate of the generated DAG species. DAGs produced by ATGL and ABHD5 are a substrate for HSL to generate FAs and glycerol as energy substrates [72]. In times of adequate energy supply, however, DAGs are re-esterified to TAGs by diacylglycerol-O-acyltransferase 2 (DGAT2) or used for the synthesis of glycerophospholipids as important membrane components [86]. Thus, the reduced formation of DAGs in NLSDI cells due to low ATGL activity impairs their use in glycerophospholipid synthesis and remodeling, as demonstrated by the studies of Igal and Coleman [58,62].

## 6. ABHD5 Interacts with LD-Associated Proteins

Numerous studies focused on the intracellular localization and regulation of ABHD5 protein interactions during lipolysis. In this context, the presence of PLINs (PLIN1—5) was demonstrated to play an essential role in lipid homeostasis as these LD-associated proteins regulate the accessibility of ABHD5 for ATGL activation in different tissues [87]. PLIN1—5 belong to the family of proteins characterized by the presence of a PAT (for **p**erilipin, **a**dipophilin, and **T**IP47) domain. This structural part of the proteins is located in the amino terminal half and includes three of six phosphorylation sites, an acidic loop, and two of three hydrophobic regions, which are suggested to target the proteins to LDs [88]. With the exception of PLIN4, all PLIN proteins exhibit protein interaction with ABHD5 [3,11,12,13,14,15,16].

**Figure 5:**
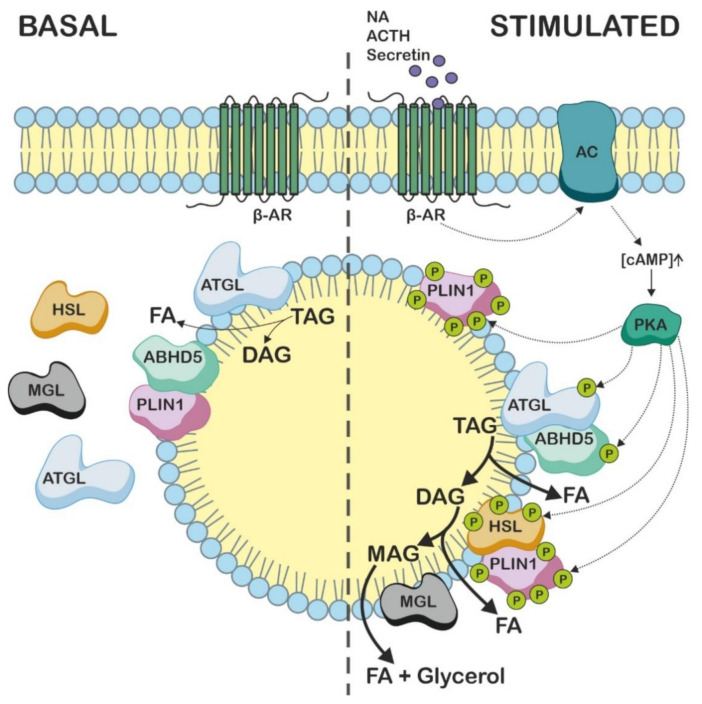
Schematic representation of basal and stimulated lipolysis in adipose tissue. Under basal conditions, perilipin 1 (PLIN1) binds α/β hydrolase domain containing protein 5 (ABHD5) and prevents the interaction of ABHD5 with adipose triglyceride lipase (ATGL), thereby suppressing lipolysis. ATGL, hormone-sensitive lipase (HSL), and monoacylglycerol lipase (MGL) are primarily located in the cytosol; however, low levels of ATGL are associated with lipid droplets and enable basal lipolysis. Activation of β-adrenergic receptors (β-AR) by noradrenalin (NA), adrenocorticotropic hormone (ACTH), or secretin leads to increased adenylate cyclase (AC) activity, resulting in an increase in cAMP levels. cAMP activates protein kinase A (PKA), which phosphorylates PLIN1, ATGL, ABHD5, and HSL. The phosphorylation of PLIN1 releases phosphorylated ABHD5, which then interacts with phosphorylated ATGL to stimulate triacylglycerol (TAG) degradation. Moreover, phosphorylated PLIN1 recruits phosphorylated HSL to the surface of lipid droplets, which hydrolyzes diacylglycerol (DAG) to monoacylglycerol (MAG). In the final step of lipolysis, MGL releases the last remaining fatty acid (FA) from the glycerol backbone.

PLIN1 (also designated as perilipin A) is the predominant paralogue in adipose tissue, regulating lipolysis [3,11,89]. PLIN1 binds ABHD5 in the basal state of lipolysis on the surface of LDs, thereby preventing the protein interaction of ABHD5 with ATGL and suppressing lipolysis [3]. During fasting or exercise, the hormonal stimulation of β-adrenergic receptors on the plasma membrane of adipocytes activates cyclic AMP-dependent protein kinase A (PKA), which subsequently phosphorylates ATGL, HSL, PLIN1, and ABHD5 [71]. These phosphorylation events interrupt the PLIN1–ABHD5 interaction, resulting in the release of ABHD5, which then activates ATGL [17]. Interestingly, the phosphorylation of ABHD5 at Ser239 by PKA increases the availability of ABHD5 to bind to ATGL, but it does not affect the co-activation mechanism per se [90]. Finally, phosphorylated PLIN1 promotes DAG hydrolysis by recruiting phosphorylated HSL to the LD surface (Figure 5) [91].

The protein interaction of PLIN1 with ABHD5 was proposed to involve the carboxyl terminus of PLIN1 consisting of an α/β domain and a four-helix bundle [3]. A study identifying human frame-shift mutations (Leu-404fs and Val-398fs) in the *PLIN1* gene provides strong evidence for this hypothesis [92]. The authors showed that the carboxyl terminal truncation of PLIN1 impairs the binding of the protein to ABHD5 and destabilizes the localization of ABHD5 to LDs, resulting in increased basal lipolysis. As a consequence, the affected patients suffer from partial lipodystrophy, hypertriglyceridemia, severe insulin resistance, and type 2 diabetes.

PLIN2 (also known as adipophilin or adipose differentiation-related protein; ADRP) is ubiquitously expressed, including adipose tissue [93]. However, unlike PLIN1, PLIN2 plays a less prominent role in regulating adipose tissue lipolysis. PLIN2 is more permissive for lipolysis and facilitates the constitutive transport of FAs in tissues that require a constant FA supply. Patel et al. [14] reported that the interaction between ABHD5 and PLIN2 is significantly weaker compared to the ABHD5–protein interaction with PLIN1, allowing for higher rates of lipolysis under basal conditions. In addition, PLIN2 does not serve as a substrate for PKA, resulting in comparable lipolysis rates under basal or stimulated conditions [91]. Consistent with these findings, *Plin2* knock-out mice display a relatively mild phenotype on a chow diet but are protected from obesity on a high-fat diet, likely due to the browning of white adipose tissue [94,95,96]. Moreover, these animals showed reduced hepatic TAG storage and disturbed mammary gland development and lactation [94,95,96,97,98]. In contrast to whole-body knock-out, liver-specific PLIN2-deficient mice are not protected against hepatic steatosis when fed a high-fat diet, suggesting a systemic hepatoprotective mechanism of PLIN2 [99]. Taken together, these studies suggest that PLIN2 serves as a lipolytic barrier to regulate FA flux in various tissues by moderately attenuating lipolysis.

PLIN3 (also known as the tail-interacting protein of 47 kDa; TIP47) is ubiquitously expressed, including white and brown adipose tissues, and shares the highest sequence similarity with PLIN2 [88,100]. In adipocytes, PLIN3 localizes to small nascent LDs, but is replaced by PLIN2 over time when the size of the LD increases [101]. In non-adipose cells, small LDs are coated with PLIN2, PLIN3, or both proteins [87,88]. Mechanistically, the role of PLIN3 in lipolysis regulation is poorly understood and has never been directly investigated. Compared to PLIN1, the interaction between PLIN3 and ABHD5 is significantly weaker, suggesting that PLIN3, like PLIN2, might be more permissive to lipolysis [14]. In line with this finding, the genetic knock-down of *Plin3* mRNA expression in AML12 cells does not alter lipolysis because LDs are instead covered with PLIN2 to compensate for the absence of PLIN3 [102]. However, the downregulation of *Plin3* expression affects the size and distribution of LDs, suggesting additional surfactant properties of the protein [102]. To gain more insight into the function of PLIN3, several in vivo studies have been performed in mice. Antisense oligonucleotide (ASO)-mediated *Plin3* transcript knock-down in mice revealed reduced TAG levels in the liver and serum, indicating the functional role of PLIN3 in stabilizing hepatic TAG stores [103]. More recently, Lee et al. [100] reported that PLIN3 may play a role as a negative regulator of thermogenesis. *Plin3* knock-out mice are cold-tolerant and display increased beige adipocyte formation in inguinal white adipose tissue. The genetic deletion of *Plin3* promotes basal and stimulated lipolysis in adipocytes, which then leads to the induction of the peroxisome proliferator-activated receptor (PPAR)-α, a transcription factor of thermogenic gene expression. Consequently, PLIN3-deficient mice showed enhanced PPARα-target gene and uncoupling protein 1 (UCP-1) expression in inguinal white adipose tissue upon cold exposure. Whether the PLIN3–ABHD5 protein interaction plays a central role in regulating thermogenesis requires further investigation. In summary, these data suggest that PLIN3 is involved in the regulation of hepatic TAG storage and prevents unnecessary energy burning by limiting thermogenic gene expression in adipose tissue.

In oxidative tissues, such as the heart, skeletal muscle, liver, or brown adipose tissue, PLIN5 (also known as muscle LD protein; MLDP, LSDP5, OXPAT, or PAT-1) is the predominant perilipin paralogue regulating lipolysis [104]. PLIN5 modulates TAG turnover by recruiting HSL onto LDs and interacting with ATGL and its co-activator ABHD5 in a PKA-dependent manner [15,16]. In the basal state of lipolysis, PLIN5 binds both ATGL and ABHD5, but the binding cannot occur simultaneously [15,16]. Granneman et al. [15] showed that in the absence of ABHD5, the targeting of ATGL to PLIN5 is insufficient to promote cellular lipolysis. Moreover, PLIN5 variants that prevent the proper targeting of ABHD5 or ATGL to LDs largely reduce TAG hydrolysis, even when both proteins are present. The authors concluded that different PLIN5 molecules form an oligomeric complex and coordinate the interaction of ATGL and ABHD5 on the surface of LDs [15]. Several in vivo studies suggest that PLIN5 protects cells from oxidative stress and mitochondrial dysfunction [105,106,107,108]. PLIN5-deficient mice lack detectable LDs in cardiomyocytes and exhibit increased FA oxidation rates [105]. As a consequence, these animals suffer from enhanced reactive oxygen species production in the heart, indicating an important cardioprotective function of PLIN5. As expected, the cardiac muscle-specific overexpression of PLIN5 results in massive TAG accumulation in the heart, which is comparable with cardiac steatosis in individuals with ATGL deficiency [109,110]. Despite this massive TAG accumulation in the heart, however, *Plin5*-transgenic mice exhibit a normal heart function and have a normal lifespan [111]. This phenotype is entirely different from that of ATGL-deficient mice, which suffer from severe cardiac dysfunction and premature death [110]. Pollak and colleagues [112] have investigated the phenotypic differences between these two mouse models in more detail. The authors reported an increased PKA expression in cardiac muscle-specific *Plin5*-transgenic mice that restores FA release from PLIN5-coated LDs. This compensatory mechanism may explain, at least in part, the normal cardiac function and lifespan of these animals despite massive cardiac TAG accumulation. A unique feature of PLIN5 is the ability to recruit mitochondria to the surface of LDs (LD–mitochondria coupling) via a 20-amino-acid stretch at the carboxyl terminus of the protein [113]. Several studies have proposed that this LD–mitochondria coupling is required to increase the FA flux from LDs to the mitochondria, allowing for an efficient FA oxidation [113,114,115,116]. This view has been challenged by a recent publication by Kien et al. [117], demonstrating that LD–mitochondria coupling does not have a significant effect on TAG turnover or β-oxidation but rather increases the mitochondrial respiratory capacity and metabolic flexibility of cardiomyocytes upon lipid loading.

The activation of lipolysis in adipose tissue and oxidative tissues largely depends on the phosphorylation of PLIN proteins and HSL by the enzymatic activity of PKA. Recently, Granneman and co-workers identified synthetic ligands which rapidly disrupt the protein interaction of ABHD5 with PLIN1 or PLIN5 [19]. These compounds directly bind ABHD5 and promote adipose tissue and muscle lipolysis in a PKA-independent manner. In contrast, long-chain acyl-CoAs have been identified as endogenous allosteric ligands of ABHD5 that reverse the effect that is induced by these synthetic ligands and suppress lipolysis by promoting the protein interaction between ABHD5 and PLIN proteins. Furthermore, acyl-CoAs have also been shown to interact with the amino terminal domain of ATGL and inhibit its catalytic activity, presumably representing product inhibition. In line with such acyl-CoA/FA-mediated product inhibition, Hofer et al. [18] showed that the presence of the FA-binding protein 4 (FABP4) stimulates ATGL-catalyzed TAG hydrolysis in an ABHD5-dependent manner. The authors demonstrated that adipocyte-type FABP interacts with ABHD5 and sequesters FAs from the first and second step of lipolysis. This protein–protein interaction prevents the product inhibition of ATGL and HSL. Whether the protein interaction of ABHD5 with other members of the FABP family is important for lipid homeostasis in tissues other than adipose tissues has not been studied so far.

## 7. Studies Using *Abhd5* Transgenic Mice Show That ABHD5 Plays an Important Role in Adipose and Non-Adipose Tissues

The generation of mice with a targeted disruption of *Abhd5* has enabled the study of ABHD5 function in lipid and energy metabolism and of its role in the pathogenesis of NLSDI in vivo [118]. Similar to patients, the whole-body knock-out of *Abhd5* in mice resulted in systemic TAG accumulation, Jordans’ anomaly, and severe hepatic steatosis, demonstrating the critical role of ABHD5 in ATGL-mediated TAG hydrolysis in vivo. Newborn mutant mice were smaller than their wild-type littermates and showed reduced circulating glucose and lipid levels. In addition, they were born with an ichthyosiform skin phenotype that, in contrast to humans, led to the premature death of the animals. To circumvent the limitations arising from the early death of global ABHD5-deficient mice, studies have been performed using ASOs to inhibit ABHD5 protein expression in adult mice or with tissue-specific *Abhd5* knock-out mice.

ASO-mediated *Abhd5* transcript knock-down mostly in the liver and white adipose tissue of adult mice did not induce obesity but instead protected the mice from high-fat diet-induced obesity and resulted in smaller epididymal fat pads due to defective adipogenesis caused by the reduced expression of lipogenic genes [119,120]. Although *Abhd5* knock-down caused hepatic steatosis linked to impaired hepatic TAG catabolism and drastically elevated levels of TAGs, DAGs, and ceramides in the liver, the mice did not develop insulin resistance. This phenomenon has been explained by the potential role of ABHD5 in generating signaling molecules that maintain the balance between inflammation and insulin signaling [121] and by sequestering the accumulating DAGs to the LD/endoplasmic reticulum (ER) fraction, thereby preventing the induction of insulin resistance through the action of PKCε [120]. In addition, the inhibition of ABHD5 protein expression by ASOs also resulted in the reduced hepatic secretion of very low-density lipoproteins (VLDLs) [4,122].

The latter finding was challenged by the phenotype of mice lacking functional ABHD5 specifically in the liver [123]. While these liver-specific ABHD5-deficient mice similarly developed hepatic steatosis associated with decreased hepatic TAG hydrolase activity and reduced FA oxidation rates, they exhibited plasma TAG concentrations and VLDL production rates comparable to their wild-type littermates. This discrepancy in the mouse phenotype of ASO-treated vs. liver-specific ABHD5-deficient mice suggests that hepatic ABHD5 does not directly influence VLDL production but rather that the ASO-mediated knock-down of the ABHD5 function in non-hepatic tissues (e.g., adipose tissue) affects hepatic VLDL secretion. Evidence for this hypothesis provides the phenotype of mice lacking functional ABHD5 specifically in adipose tissue [124]. These mice exhibit markedly reduced levels of circulating energy substrates under fasting conditions, including TAGs and FAs. Whether VLDL secretion rates are impaired in this mouse model remains elusive, but Jaeger et al. [124] found that defective adipose tissue lipolysis decreased PPARα signaling and the nuclear translocation of the cAMP-responsive element binding protein (CREB) in the liver. This severely reduced the target gene expression of the ATGL inhibitor G0S2 and, as a consequence, impaired hepatic TAG metabolism and systemic energy homeostasis. The administration of lipids and raising circulating FA levels reversed the liver phenotype in fasted adipose tissue-specific *Abhd5* knock-out mice, indicating that adipose tissue-derived FAs control hepatic TAG breakdown.

The role of ABHD5 in muscle energy metabolism has been investigated by Zierler et al. [125] using muscle-specific ABHD5-deficient mice. The authors of this study showed that the loss of functional ABHD5 in mouse myocardium led to cardiac myopathy associated with defective TAG catabolism and impaired mitochondrial FA oxidation. Yet, this phenotype was milder than in *Atgl* knock-out animals, but also affected PPARα signaling, supporting the hypothesis that ATGL-mediated TAG hydrolysis generates a signaling molecule that directly or indirectly regulates the mitochondrial function via PPARα-regulated target gene expression. These findings have been corroborated by a study by Xie and colleagues [126]. The authors additionally showed that mice with a muscle-specific loss of ABHD5 function exhibited increased glucose tolerance and insulin sensitivity on a high-fat diet, which was accompanied by TAG accumulation in both the heart and oxidative skeletal muscle. Similar findings were observed in studies using human primary myotubes, in which the knock-down of *ABHD5* expression resulted in reduced TAG hydrolysis and decreased release and oxidation of TAG-derived FAs [127].

More recently, Jebessa et al. [128] demonstrated that during cardiac lipolysis, ABHD5 not only stimulates ATGL activity, but also cleaves the signal-responsive epigenetic regulator histone deacetylase 4 (HDAC4). The resulting amino terminal HDAC4 polypeptide then represses myocyte enhancer factor 2 (MEF2)-dependent gene expression, protecting mice and humans from heart failure. Remarkably, this proteolytic cleavage of HDAC4 represents the first report that ABHD5 acts as a protease. However, since the murine and human protein lack the serine in the putative active site, this would suggest that ABHD5 is not a classical serine protease and that other amino acid residues replace the catalytic serine in its nucleophilic elbow. Furthermore, this proteolytic activation of HDAC4 would imply that other binding partners of ABHD5 may also require proteolytic cleavage for their full enzymatic activity.

Xie et al. reported on the intestine-specific role of ABHD5 in mice [129]. They showed that intestinal ABHD5 deficiency results in reduced TAG hydrolase activity and massive TAG accumulation in enterocytes. Consequently, postprandial plasma TAG concentrations are reduced in this mouse model, implicating that ABHD5 is required for efficient postprandial intestinal chylomicron secretion. These findings were challenged by the phenotype of mice lacking both ABHD5 and ATGL specifically in the intestine [130]. These double knock-out mice exhibited unchanged plasma parameters and comparable chylomicron secretion rates, which had been explained by the fact that the diet used in this study had a different composition than that used by Xie and colleagues [129]. Yet, the authors have shown that ABHD5, together with ATGL, is involved in the catabolism of intestinal LDs formed during the re-absorption of lipids from the basolateral side of enterocytes but not in the hydrolysis of apically derived lipids involved in chylomicron formation.

The function of ABHD5 in the lipid and energy metabolism of macrophages and its role in the immune system have been investigated in several in vivo studies by Kratky and co-workers [131,132,133]. The authors showed that the genetic ablation of *Abhd5* or *Atgl* in macrophages led to different phenotypes in mice. While the loss of ATGL function in macrophages causes mitochondrial dysfunction and ER stress due to defective lipolysis, this phenotype was not observed in macrophages lacking functional ABHD5 [131,132,134]. This discrepancy in the phenotypes was explained by basal ATGL activity in ABHD5-deficient macrophages, which may be sufficient to prevent cells from mitochondrial apoptosis and ER stress [131]. ATGL deficiency in macrophages led to the alternative M2-like phenotype associated with anti-inflammatory responses [135], resulting in the reduced development of atherosclerotic lesions in low-density lipoprotein receptor (LDLR)-deficient mice [136]. In contrast, macrophage-specific *Abhd5* deletion was associated with reduced PPARγ signaling and overproduction of reactive oxygen species [137]. As a response, macrophages lacking functional ABHD5 acquire a “pro-atherogenic” M1-like phenotype, which has been shown to activate the nucleotide-binding domain, leucine-rich-containing family, pyrin domain-containing-3 (NLRP3) inflammasome, leading to the release of pro-inflammatory cytokines [131,137]. Consistent with this finding, the macrophage-specific overexpression of *Abhd5* reduced atherosclerotic plaque formation in apolipoprotein E (apoE)-deficient mice [138]. Collectively, these data of mutant mouse models strongly suggest distinct roles of ATGL and ABHD5 in macrophage function and atherosclerosis development.

## 8. ABHD5 Possesses an ATGL-Independent Function in the Skin

Since ATGL and ABHD5 act in the same biochemical pathway, the inactivation of either protein is expected to result in comparable clinical phenotypes that resemble NLSD. However, only mutations in the *ABHD5* gene are linked to the pathogenesis of ichthyosis (NLSDI) [24]. This rare genodermatosis belongs to the heterogeneous group of Mendelian disorders of cornification and is characterized by an excessive and very visible scaling, usually affecting the entire skin’s surface [139,140,141]. As is typical for ichthyosis, the affected patients suffer from a disturbed skin barrier function and, as a result, an increased susceptibility to bacterial infections, but also to allergic diseases such as asthma [142,143]. In order to normalize the barrier function, the skin drives epidermal hyperplasia resulting in the formation of multiple layers of corneocytes (hyperkeratosis) and abnormal desquamation [139]. Interestingly, these clinical features of ichthyosis or other skin abnormalities have not been reported in NLSDM patients [49] or in ATGL-deficient mice [110], arguing for an ATGL-independent function of ABHD5 in skin physiology.

The first evidence of the keratinocyte-specific function of ABHD5 was provided by our laboratory in characterizing the skin of mice that globally or epidermis-specifically lacks functional ABHD5 [118,144]. Similar to NLSDI patients, the loss of functional ABHD5 in mice results in an ichthyosiform skin phenotype associated with a defective permeability barrier. However, the skin barrier defect is more severe in mice than in humans, because mice die within hours after birth due to rapid dehydration. This finding is consistent with results from several other mouse models affected by an impaired skin permeability barrier [145,146,147,148,149,150,151,152] and suggests that mice are more sensitive to barrier defects than humans. A plausible explanation for this interesting species-specific phenotype could be that mice have an unfavorable body-volume-to-skin-surface-area ratio that promotes increased dehydration when the water permeability barrier is disrupted. Furthermore, human newborns may also benefit from clinical care taking that is absent in mice housing.

**Figure 6:**
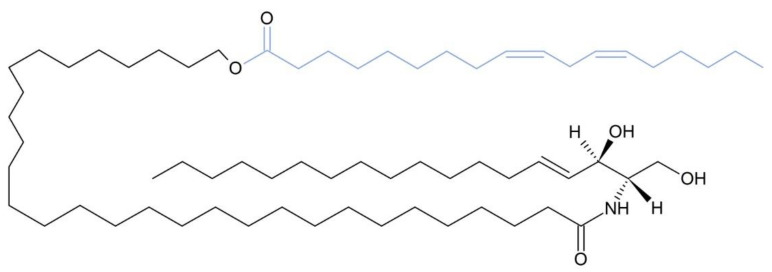
Molecular structure of ω-*O*-acylceramide. The α/β hydrolase domain containing protein 5 is involved in the esterification of ω-hydroxyceramide with linoleic acid (fatty acid chain colored in purple), producing ω-*O*-acylceramide. The molecular similarities between ω-*O*-acylceramide and triacylglycerol estolide (compared with Figure 4) are worth noting, as both molecules contain a fatty acid esterified to a hydroxy fatty acid.

ABHD5 is highly expressed in the skin in both the dermis and the epidermis [144]. In the epidermis, ABHD5 is localized in the upper layers and its expression strongly increases upon keratinocyte differentiation [144]. Consistent with a fundamental role of ABHD5 during cornification, biochemical analyses of mouse epidermis revealed that the absence of functional ABHD5 leads to a defect in the final step of ω-*O*-acylceramide (acylCer) synthesis (Figure 6) [118,144].

As a result, mouse ABHD5-deficient epidermis lacks acylCers, whereas free extractable ω-hydroxyceramides, the direct precursors of acylCers, massively accumulate. These findings conform to observations in the epidermis of NLSDI patients [153]. Using ultrastructural analyses, Peter Elias’ group as well as our laboratory showed that this defect in acylCer synthesis is associated with the impaired formation of the corneocyte-bound lipid envelope in the *stratum corneum* of mice and humans [144,153]. The loss of this epidermal structure typically compromises skin integrity and permeability barrier homeostasis [154], resulting in increased transepidermal water loss and rapid dehydration, as demonstrated in ABHD5-deficient individuals [118,144,155]. Moreover, pulse-chase experiments performed with mouse epidermal explants and radiolabeled FAs as tracer provided strong evidence that ABHD5 is involved in the transfer of essential FAs from epidermal TAG stores to ω-hydroxyceramides, thereby generating acylCers [118]. Considering these facts, the data obtained from ABHD5-deficient epidermis led to two possible hypotheses for the function of ABHD5 in epidermal lipid metabolism: i) ABHD5 itself catalyzes the ω-*O*-esterification step of acylCer biosynthesis or ii) ABHD5 stimulates a yet unknown ω-acyltransferase or transacylase that catalyzes this reaction. The first hypothesis according to which ABHD5 catalyzes an esterification reaction seems rather unlikely since the initially reported LPAAT activity of ABHD5 [25,26] has been refuted and attributed to the bacterial contamination of the ABHD5 preparations (see above) [27]. Thus, available evidence does not favor the first hypothesis that ABHD5 harbors an acyltransferase activity catalyzing the ω-*O*-esterification reaction for acylCer formation. Instead, it appears more plausible that, according to the second hypothesis, ABHD5 is indirectly involved in the final step of acylCer biosynthesis [144].

## 9. ABHD5 Co-Activates PNPLA1 Function for Efficient Synthesis of AcylCers

The enzyme that catalyzes the final step of acylCer biosynthesis remained undiscovered until recently. Important progress in the identification of this enzyme has been made when Grall and colleagues [156] reported mutations in the ATGL-homologous *PNPLA1* gene in humans and golden retriever dogs. Consistent with the predominant expression of PNPLA1 in the granular layer and its strong upregulation during keratinocyte differentiation, the authors found that mutations in *PNPLA1* are associated with the pathogenesis of ichthyosis in humans and dogs. Affected individuals show symptoms of fine white scales and moderate erythroderma, but interestingly, the analysis of the blood smears did not reveal any neutral lipid-containing vacuoles in granulocytes, as typically present in NLSDI or NLSDM patients. Following this initial report, several other research groups identified *PNPLA1* mutations in humans linked to ichthyosis pathogenesis [157].

In contrast to other PNPLA family members, the functional role of PNPLA1 has been enigmatic until recently. When the *PNPLA1* gene was first identified on the chromosome 6p21.31 using bioinformatics, the corresponding amino acid sequence lacked part of the amino terminal domain including the active site serine, making it unlikely to function as a lipase [70]. Wilson et al. [66] later identified exon 1 of *PNPLA1* containing the lipase/esterase GxSxG motif. Furthermore, tissue expression profiling in mice and humans revealed low levels of mRNA transcripts in the digestive tract (stomach, small intestine), bone marrow, and spleen, in addition to high levels in the skin [66,158,159]. The bioinformatic and functional analyses by Chang and colleagues [160] identified mouse PNPLA1 as a membrane-associated protein with multiple putative transmembrane domains. In addition, a genetic study summarizing the localization of previously known *PNPLA1* mutations in ichthyosis patients concluded that PNPLA1, like ATGL, requires a larger region than the core patatin domain to exert its full enzymatic activity [161]. However, unlike its family members, no enzymatic activity or physiological function could be attributed to PNPLA1. To gain insight into the underlying molecular mechanisms that cause PNPLA1 ichthyosis pathogenesis, our laboratory and two other laboratories independently characterized mice lacking functional PNPLA1 [159,162,163]. Interestingly and identical to mice with a homozygous disruption of the *Abhd5* gene, PNPLA1-deficient mice suffered from a severe skin permeability barrier defect. Again, increased transepidermal water loss resulted in the premature death of the animals within a few hours after birth. Functional analyses of both the epidermis of PNPLA1-deficient mice and of primary keratinocytes from patients with mutant *PNPLA1* revealed a defect in acylCer biosynthesis very similar to that found in humans and mice lacking ABHD5 function [159]. The absence of functional PNPLA1 also drastically reduces the epidermal amount of acylCers, whereas ω-hydroxyceramides specifically accumulate in the epidermis. This characteristic defect in epidermal sphingolipid metabolism caused by the absence of functional PNPLA1 suggested that PNPLA1 is the unknown enzyme that catalyzes the esterification of ω-hydroxyceramides with linoleic acid. Consistent with this hypothesis, PNPLA1 has recently been found to function as a transacylase that uses linoleic acid-containing TAG as acyl donor to produce acylCers for an intact skin permeability barrier [164]. Yet, the reported transacylase activity of PNPLA1 is relatively low compared to other members of the PNPLA family [69,82,84,165], which suggested that PNPLA1 may require additional proteins or factors to reach full enzymatic activity [159]. Indeed, functional studies in our laboratory showed that ABHD5 directly interacts with PNPLA1 and recruits the enzyme to its putative TAG acyl donor substrate onto LDs [8]. This protein translocation in the presence of ABHD5 strongly stimulated the esterification of ω-hydroxyceramides with linoleic acid, indicating that ABHD5 acts as a co-activator of PNPLA1 for the synthesis of acylCers. Conversely, mutant ABHD5 associated with NLSDI in humans failed to accelerate PNPLA1-mediated acylCer biosynthesis [8,166]. These findings demonstrate for the first time an essential, ATGL-independent function of ABHD5 in skin barrier physiology and provide a plausible explanation for the molecular mechanism underlying ichthyosis pathogenesis in NLSDI patients. However, the exact mechanism of activation of PNPLA1 by ABHD5 remains to be determined but may also require the amino acid residues of ABHD5 to be involved in the interaction of the protein with ATGL.

## 10. ATGL and ABHD5 Exhibit Distinct Functions in Hepatic Lipid Metabolism and Inflammation

Defective neutral lipid storage is often associated with the pathogenesis of non-alcoholic fatty liver disease (NAFLD) in humans [48,167]. The clinical condition of this disorder includes massive hepatic lipid accumulation (also known as hepatosteatosis) that occurs in the absence of viral infections or excessive alcohol consumption. NAFLD often manifests as asymptomatic steatosis or inflammatory steatohepatitis (non-alcoholic steatohepatitis; NASH), which may further progress into fibrosis, cirrhosis, or eventually hepatocellular carcinoma [167]. Interestingly, NLSDI patients typically suffer from progressive fatty liver disease associated with splenomegaly [47]. Similarly, the liver-specific deletion of *Abhd5* in mice results in pronounced hepatosteatosis that progresses with age into NASH and fibrosis [123]. This hepatic phenotype is more pronounced in liver-specific *Abhd5* knock-out mice than in mice lacking ATGL globally or specifically in hepatocytes [110,124,125]. The latter develop mild liver steatosis, but this never progresses into inflammatory liver disease, supporting the hypothesis that ABHD5 regulates hepatic neutral lipid storage and inflammation via mechanisms not involving ATGL. In order to investigate the ATGL-independent functions of ABHD5 in hepatic lipid and energy metabolism, Lord et al. [168] used ASOs to selectively knock-down ABHD5 protein expression in the liver of wild-type or global *Atgl* knock-out mice. The authors demonstrated that the lack of functional ABHD5 causes hepatic steatosis in the presence or absence of ATGL. This observation underlines the fact that the regulation of lipolysis in non-adipose tissues differs from the catecholamine-stimulated mechanism in adipocytes. Moreover, the genetic deletion of *Atgl*, but not *Abhd5*, resulted in reduced de novo lipogenesis in the liver and adipose tissue of mice [168]. Consistent with the phenotypic differences between individuals lacking functional ATGL or ABHD5, the study confirmed that ABHD5 suppresses inflammatory responses in the liver, suggesting distinct roles of ATGL and ABHD5 in the control of hepatic inflammation. Taken together, these findings clearly demonstrate that ABHD5 regulates lipid homeostasis and inflammation independently of ATGL in the liver, although the underlying molecular mechanisms remain to be further elucidated.

## 11. Protein Interaction of ABHD5 with PNPLA3 Is Critical in the Development of Fatty Liver Disease

Important progress in understanding the ATGL-independent functions of ABHD5 in hepatic lipid and energy metabolism has been made when a *PNPLA3* gene variant was associated with the pathogenesis of fatty liver disease in patients. The human *PNPLA3* gene, also named adiponutrin, is located on chromosome 22q13.31 and spans nine exons with a transcript length of 2805 base pairs. The protein comprises 481 amino acids and shares the highest sequence identity with ATGL [65,169]. Human PNPLA3 is mainly expressed in the retina and liver, where it intracellularly localizes to LDs [170]. The protein exhibits a predominant TAG hydrolase activity, but also shows acylglycerol transacylase, and weak acyltransferase as well as retinyl ester hydrolase activity [69,70,170,171,172]. The physiological role of PNPLA3 in lipid metabolism has long been unclear because neither the overexpression nor the deletion of *Pnpla3* in mice resulted in specific phenotypes [169,173]. The identification of the *PNPLA3* variant rs738409, which encodes for an amino acid substitution of isoleucine to methionine at position 148 (p.Iso148Met) of the protein in patients with alcoholic or non-alcoholic fatty liver disease, shed important light on the enigmatic role of PNPLA3 in hepatic lipid and energy metabolism [174,175,176,177]. This mutant variant shows markedly reduced TAG hydrolase activity, suggesting that fatty liver disease in affected patients is caused by a loss of enzymatic function [171]. However, this hypothesis is in sharp contrast to the phenotype of PNPLA3-deficient mice, which show no signs of fatty liver disease [169,173], whereas the overexpression of defective PNPLA3 variants (p.Iso148Met or p.Ser47Ala) leads to hepatic steatosis in mice [178,179]. The PNPLA3 (p.Iso148Met) variant accumulates on the surface of LDs by evading ubiquitylation and proteasomal degradation [180]. These findings raised the question of how an accumulation of defective PNPLA3 on LDs causes increased lipid levels in hepatocytes. Several groups have hypothesized that the PNPLA3 (p.Iso148Met) variant increases TAG synthesis or impairs TAG secretion from the liver, but these theories have not been supported by evidence [172,181]. Recently, two groups independently reported that the pathogenesis of hepatic steatosis in carriers of the PNPLA3 (p.Iso148Met) variant is an ABHD5-dependent process [9,10]. The authors clearly demonstrated that PNPLA3 competes with ATGL for binding to ABHD5, thereby suppressing ABHD5-dependent lipolysis. The protein interaction between the PNPLA3 (p.Iso148Met) variant and ABHD5 is even stronger than with ATGL, limiting the availability of ABHD5 for ATGL activation [10]. Decreased ATGL-mediated lipolysis subsequently led to reduced TAG degradation and increased fat accumulation in the liver [9]. In summary, these findings suggest that ATGL and PNPLA3 have opposing functions in TAG metabolism, at least in the liver. Future studies need to clarify how the correct stoichiometric ratio of PNPLA3, ATGL, and ABHD5 is maintained to regulate LD homeostasis in hepatocytes.

## 12. Concluding Remarks

ABHD5 has been shown to be an indispensable regulator of lipid metabolism that affects lipid and energy homeostasis in various tissues of the body. Its ability to interact with different proteins and enzymes in a cell-type specific manner and to affect their enzymatic activity or function is intriguing. In addition to the initially identified ABHD5–ATGL protein interaction, recent studies have provided important new insights into the ATGL-independent functions of ABHD5. The genetic deletion of *Abhd5* in mice has uncovered that ABHD5 affects lipid homeostasis not only in adipose tissue but also in liver and skin by cooperating with other PNPLA proteins that are homologous to ATGL. Future studies are required to further explore these ATGL-independent roles of ABHD5 in the context of, for example, hepatic and epidermal lipid metabolism, but also intestinal lipid uptake and the immune system. Furthermore, to date, it is unknown which processes coordinate the different regulatory roles of ABHD5 with its multiple binding partners and whether different amino acid residues of ABHD5 are involved in these protein–protein interactions. The findings of such studies will shed light on whether ABHD5-protein interactions are facilitated by the same interaction surface or whether different binding partners interact at the same time and are co-activated, proteolytically processed, and/or change their intracellular localization. The availability of an experimental three-dimensional model of ABHD5 and a co-crystallization with its binding partners would be of significant help to answer these questions. Such a three-dimensional model would also allow for the exploration of the molecular basis of how ABHD5 facilitates the co-activation of its binding partners and how it interacts with the lipid intermediates involved. The findings from such studies would even allow for the exploration and prediction of whether and how ABHD5 exerts additional catalytic activities. Along that line, studies specifically designed to elucidate whether and/or how these potential enzymatic activities of ABHD5 affect its ability to interact with its binding partners would be required. The answers to these questions will greatly advance our understanding of the ABHD5 protein’s function and its role in regulating lipid and energy metabolism in humans.

## Abbreviations

ABHD5, α/β hydrolase domain-containing protein; acylCer, ω-*O*-acylceramide; ATGL, adipose triglyceride lipase; ASO, antisense oligonucleotide; CDS, Chanarin-Dorfman syndrome; CGI-58, comparative gene identification-58; DAG, diacylglycerol; ER, endoplasmic reticulum; FA, fatty acid; FABP, fatty acid-binding protein; FAHFA, fatty acid ester of hydroxy fatty acid; G0S2, G0/G1 switch gene 2; HDAC4, histone deacetylase 4; HSL, hormone-sensitive lipase; LD, lipid droplet; LPAAT, lysophosphatidic acid acyltransferase; MAG, monoacylglycerol; MGL, monoacylglycerol lipase; NAFLD, non-alcoholic fatty liver disease; NASH, non-alcoholic steatohepatitis; NLSDI/M, neutral lipid storage disease with ichthyosis/myopathy; PLIN, perilipin; PNPLA, patatin-like phospholipase domain-containing; PKA, protein kinase A; PPAR; peroxisome proliferator-activated receptor; TAG, triacylglycerol; VLDL, very low-density lipoprotein.
